# Study of the Mechanism of Astragali Radix in Treating Type 2 Diabetes Mellitus and Its Renal Protection Based on Enzyme Activity, Network Pharmacology, and Experimental Verification

**DOI:** 10.3390/molecules28248030

**Published:** 2023-12-10

**Authors:** Chunnan Li, Kaiyue Zhang, Lu Liu, Jiaming Shen, Yuelong Wang, Yiying Tan, Xueqin Feng, Wanjie Liu, Hui Zhang, Jiaming Sun

**Affiliations:** 1 Jilin Ginseng Academy, Changchun University of Chinese Medicine, Changchun 130117, China; lcn1013@hotmail.com (C.L.); zky960523@163.com (K.Z.); 15804426590@163.com (L.L.); sjm1836778759@163.com (J.S.); wyuelong1994@163.com (Y.W.); t2589954433@163.com (Y.T.); fxq5691@126.com (X.F.); 15948447786@163.com (W.L.); 2 Jilin Correction Pharmacy New Drug Development Co., Ltd., Changchun 130012, China

**Keywords:** Astragali Radix, T2DM, network pharmacology, UPLC-QE-Orbitrap-MS, mechanism

## Abstract

Astragali Radix (AR) is a common Chinese medicine and food. This article aims to reveal the active role of AR in treating Type 2 diabetes mellitus (T2DM) and its renal protective mechanism. The hypoglycemic active fraction was screened by α-glucosidase and identified by UPLC-QE-Orbitrap-MS spectrometry. The targets and KEGG pathway were determined through the application of network pharmacology methodology. Molecular docking and molecular dynamics simulation technology were used for virtual verification. Subsequently, a mouse model of T2DM was established, and the blood glucose and renal function indexes of the mice after administration were analyzed to further prove the pharmacodynamic effect and mechanism of AR in the treatment of T2DM. HA was determined as the best hypoglycemic active fraction by the α-glucosidase method, with a total of 23 compounds identified. The main active components, such as calycoside-7-*O*-β-D-glucoside, methylnisoline, and formononetin, were revealed by network pharmacology. In addition, the core targets and the pathway have also been determined. Molecular docking and molecular dynamics simulation techniques have verified that components and targets can be well combined. In vivo studies have shown that AR can reduce blood sugar levels in model mice, enhance the anti-inflammatory and antioxidant activities of kidney tissue, and alleviate kidney damage in mice. And it also has regulatory effects on proteins such as RAGE, PI3K, and AKT. AR has a good therapeutic effect on T2DM and can repair disease-induced renal injury by regulating the RAGE/PI3K/Akt signaling pathway. This study provides ideas for the development of new drugs or dietary interventions for the treatment of T2DM.

## 1. Introduction

Diabetes mellitus encompasses a collection of endocrine disorders characterized by elevated blood glucose levels, leading to potential damage to bodily tissues and organs as a result of various factors [1]. Type 2 diabetes mellitus (T2DM) predominates among individuals diagnosed with diabetes mellitus, and its prevalence has steadily risen in recent years, garnering significant scholarly interest [2]. Diabetic nephropathy (DN), a severe microvascular complication of T2DM, stands as a primary contributor to end-stage renal disease, boasting a substantial global incidence and mortality rate [3]. Metformin hypoglycemic agents are commonly prescribed for the treatment of Type 2 diabetes mellitus (T2DM), but prolonged use may result in drug resistance and increased side effects [4]. In contrast, traditional Chinese medicine (TCM) possesses the attributes of being multicomponent and multitargeted, enabling a comprehensive control of blood sugar levels and the alleviation of associated complications in diabetic patients [5].

Astragali Radix (AR) is derived from the root of *Astragalus membranaceus* (Fisch.) Bge. var. *mongholicus* (Bge.) Hsiao (*A. mongholicus*) and *Astragalus membranaceus* (Fisch.) Bge. (*A. membranaceus*). Among these, Mongolian Astragalus is the prevailing species and is extensively employed in China for its qi-invigorating and blood-nourishing properties [6]. AR serves as a frequently utilized traditional Chinese medicine (TCM) and health food, finding widespread application in the clinical management of diverse systemic ailments, yielding notable clinical outcomes. Polysaccharides, glycosides, flavonoids, betaine, amino acids, folic acid, carotene, choline, linoleic acid, and other trace elements are some of the main active components of AR [7]. According to modern research, AR has anticancer, anti-inflammatory, and antioxidant properties, blood lipids, and improves immunity [8,9,10,11]. It is reported in the literature that AR is a common drug in the compound prescription for treating T2DM, and it can effectively prevent renal injury caused by T2DM [12,13,14].

However, there is a lack of comprehensive research on the active components and mechanism of AR in the treatment of T2DM. Therefore, to better understand the efficacy of AR in the treatment of T2DM and its protective mechanism against renal injury, we conducted in-depth research by means of enzyme activity, network pharmacology, mass spectrometry, molecular dynamics simulation, and animal models, aiming at clarifying the active mechanism of AR. The expected result of this study is to establish a scientific basis for developing new drugs or dietary supplements that can effectively intervene in the management of T2DM.

## 2. Results

### 2.1. Results of α-Glucosidase Inhibition

**Figure 1:**
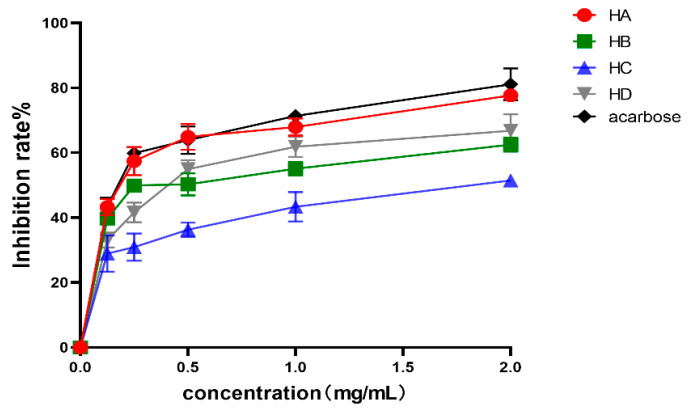
Inhibitory rate of different components of AR on α-glycosides. Values are expressed as mean ± SD (*n* = 3).

The AR aqueous decoction was subjected to membrane separation and aqueous alcohol precipitation to obtain fraction A (HA), fraction B (HB), fraction C (HC), and fraction D (HD), respectively. The inhibitory effects of different fractions of AR on α-glucosidase are evident in Figure 1. The concentration of 0.125 to 2 mg/mL demonstrates a dose–effect relationship, with the inhibitory effect increasing as the concentration increases. The IC_50_ values for HA, HB, HC, and HD on α-glucosidase were determined to be 0.18 ± 0.06 mg/mL, 0.39 ± 0.05 mg/mL, 1.96 ± 0.03 mg/mL, and 0.43 ± 0.05 mg/mL, respectively. These results indicate a statistically significant difference (*p* < 0.05). The present analysis reveals that HA exhibits the most potent inhibitory effect on α-glucosidase and comprises the most efficacious hypoglycemic active constituents of AR. It can be seen that the inhibition rate of HA is close to that of acarbose. Consequently, HA warrants further investigation in subsequent stages.

### 2.2. Analysis of HA Components

**Figure 2:**
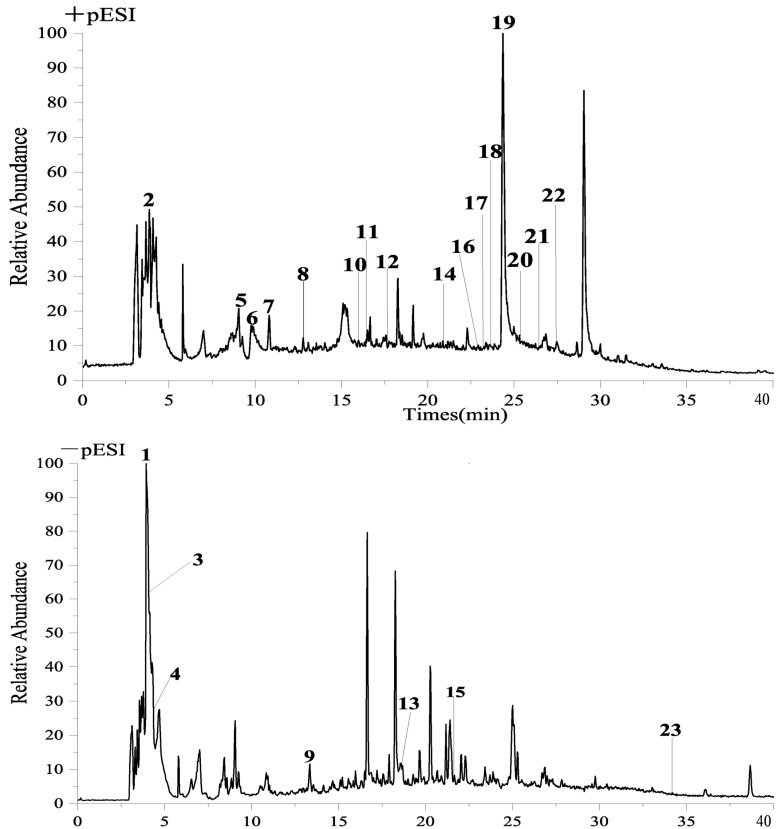
UPLCQE−Orbitrap−MS liquid quality analysis chart.

The analysis and identification of HA were conducted using UPLC-QE-Orbitrap-MS, resulting in the identification of 23 active compounds, as presented in Table 1. The ion diagram can be observed in Figure 2.

### 2.3. AR Prediction of Core Targets for T2DM Treatment

**Figure 3:**
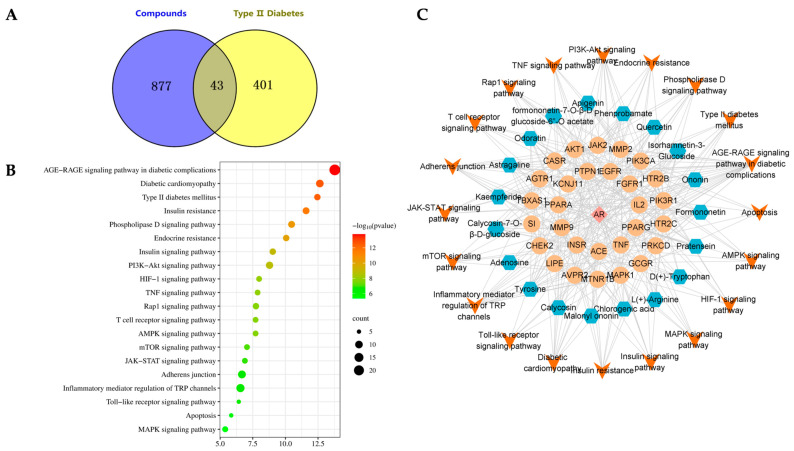
Study of network pharmacology of AR in the treatment of T2DM. (**A**) Common targets. (**B**) KEGG pathway analysis. (**C**) “Component-effect target-pathway” network.

A comprehensive analysis was conducted utilizing the Swiss Target Prediction database, resulting in the prediction of 1869 components and the exclusion of 920 targets. By conducting a search, a total of 1097 disease targets associated with T2DM were identified, which were further refined to 444 disease-related targets after merging and eliminating duplicates. The intersection of disease targets and AR potential component targets yielded 43 common targets, as represented in Figure 3A.

### 2.4. KEGG Pathway Analysis and Construction of “Component-Effect Target-Pathway” Network

**Figure 4:**
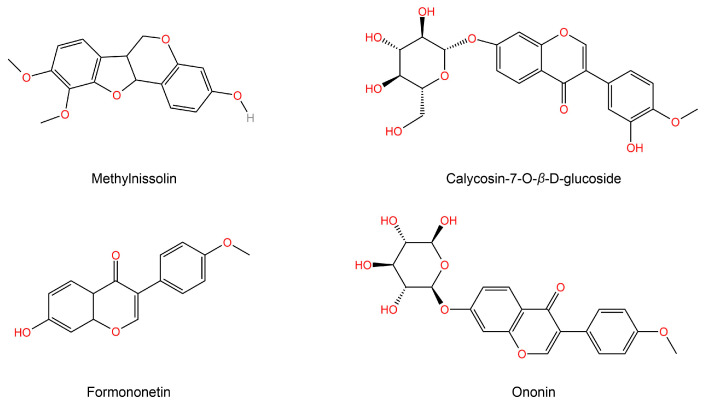
Topological parameter analysis of a network diagram obtained the top active ingredient structure of AR in treating T2DM.

The KEGG function enrichment analysis and visualization of core targets were conducted using the Metascape database. The analyzed KEGG pathways encompassed the AGE-RAGE signaling pathway in diabetic complications, Type 2 diabetes mellitus, and PI3K-Akt signaling pathway, among others. Figure 3B displays the top 20 signaling pathways with their corresponding *p* values. Cytoscape was utilized to incorporate components, core effect targets, and signaling pathways, resulting in the construction of a network diagram depicting the relationship between medicinal flavor components, core effect targets, and pathways (Figure 3C). The findings indicate that methylnissolin, calycosin-7-*O*-*β*-D-glucoside, formononetin, ononin, and other components may play a crucial role in the treatment of T2DM as key components of AR (Figure 4). Additionally, PIK3CA, AKT1, MAPK1, and TNF were identified as key targets.

### 2.5. Molecular Docking Results

**Figure 5:**
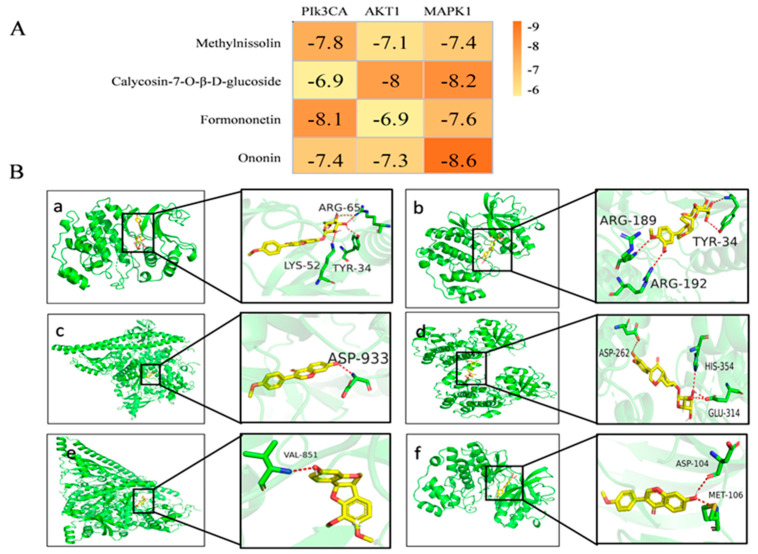
Molecular docking results and visualization. (**A**) Molecular docking results of key components with various targets. (**B**) The 3D image of the docking of components with the core target. (**a**) Ononin and MAPK1. (**b**) Calycosin-7-*O*-*β*-D-glucoside and MAPK1. (**c**) Formononetin and PIk3CA. (**d**) Calycosin-7-O-*β*-D-glucoside and AKT1. (**e**) Methylnissolin and PIk3CA. (**f**) Formononetin and MAPK1.

Three primary targets, AKT1 (PDB ID:6NPZ), MAPK1 (PDB ID:6OPH), and PI3KCA (PDB ID:4A55), identified as having the highest degree value in the network diagram, along with the top components, were chosen for molecular docking analysis. All docking results yielded negative outcomes (Figure 5A). Notably, a lower binding energy corresponded to a stronger binding ability and a more stable conformation formation. Among them, the binding energy of target AKT1 to the original ligand was only −3.1 kcal/mol, while the binding energies of MAPK1 and PI3KCA to the original ligand were −8.6 kcal/mol and −8.2 kcal/mol, respectively. Generally speaking, the molecular docking results of our ligands with the target were very good, being similar to those of the original ligands, and some results were even better than those of the original ligands. Figure 5B depicts the specific binding site of each protein and the calculated specific binding energy of each target, highlighting those with superior binding energy.

### 2.6. Molecular Dynamics Simulation

**Figure 6:**
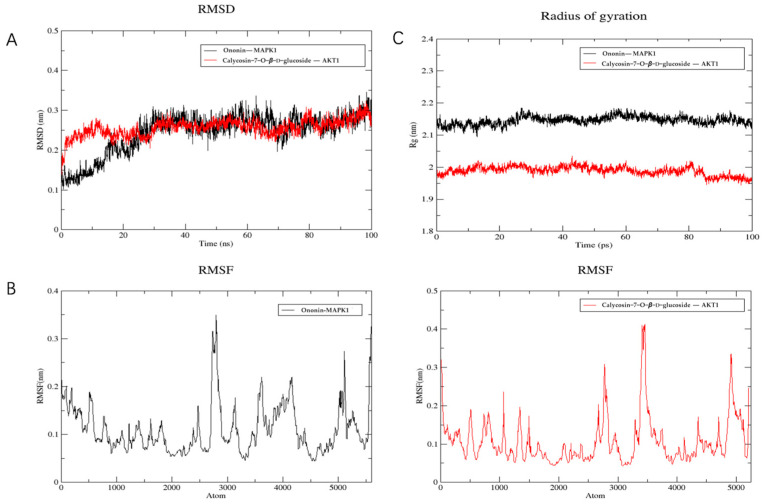
Molecular dynamics simulation. (**A**) RMSD analysis. (**B**) RMSF analysis. (**C**) Gyration radius.

The stability of the binding between the top two compounds and the target protein was confirmed through the implementation of molecular dynamics simulation. The CHARMM force field was incorporated into the system, and 100 ns simulation sampling was conducted to investigate the structural stability of the complex-target protein during the molecular dynamics simulation. The simulated trajectory was analyzed and the root mean square deviation (RMSD), root mean square fluctuation (RMSF), and protein radius of gyration (RG) were evaluated. The RMSD serves as a metric for assessing the stability of the system. During the simulation process, it was evident that the RMSD value of the complex and the target protein consistently remained at a low level. This observation suggests a strong binding affinity between the ligand and the receptor, resulting in the formation of a stable complex within a stable range (Figure 6A). Furthermore, analysis of Figure 6B reveals that the majority of receptor residues involved in the compound-target protein interaction remain within a stable range, with minimal fluctuations in the RMSF value throughout the simulation process. Finally, the radius of gyration is used to represent the compactness of the protein structure in the simulation process. According to the analysis of Figure 6C, the protein shows a smaller radius of gyration, indicating that the protein structure is more compact and the composite is more stable.

### 2.7. In Vivo Activity Verification

#### 2.7.1. The Effect of HA on the Body Weight and Blood Sugar in Model Mice

**Figure 7:**
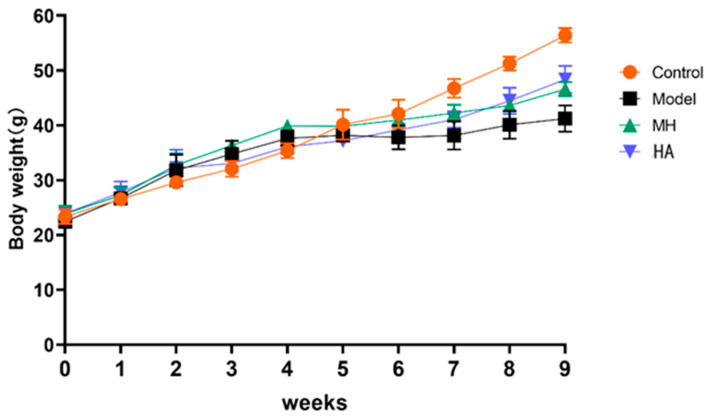
The effect of drug administration on the weights of mice over 9 weeks. Values are expressed as mean ± SD (*n* = 6).

Compared with the administration group and the model control group (Figure 7), the weight of mice in the control group increased steadily, which is normal physiological weight gain; the model group, MH group, and HA group were fed with high-fat feed for the first 4 weeks, so the weight of the mice was heavier than that of control group. At the fourth week, STZ was injected into the model. Compared with control healthy mice, the weight gain of other groups of mice decreased. After 5 weeks of administration, the weight gain of mice in the model group was slow, while the weight gain of mice in other groups increased, and it was related to time.

Following the successful implementation of the model mice, the blood glucose levels of the mice in each respective group were measured after a 4-week period of administration, as presented in Table 2. Notably, the blood glucose levels of the healthy mice in the control group exhibited no significant increase, thus lacking statistical significance. Conversely, compared to the healthy mice in the control group, the model group demonstrated a significantly elevated blood glucose level (*p* < 0.05). The blood glucose levels of mice in the MH and HA groups gradually decreased and exhibited a direct correlation after 4 weeks of treatment, compared to the model group. Furthermore, it is evident that the HA treatment group displayed a more pronounced reduction in blood glucose levels compared to the MH group, suggesting that HA exhibits a favorable hypoglycemic effect, which improves with prolonged administration.

#### 2.7.2. Effect of HA on Serum Renal Function in Model Mice

**Figure 8:**
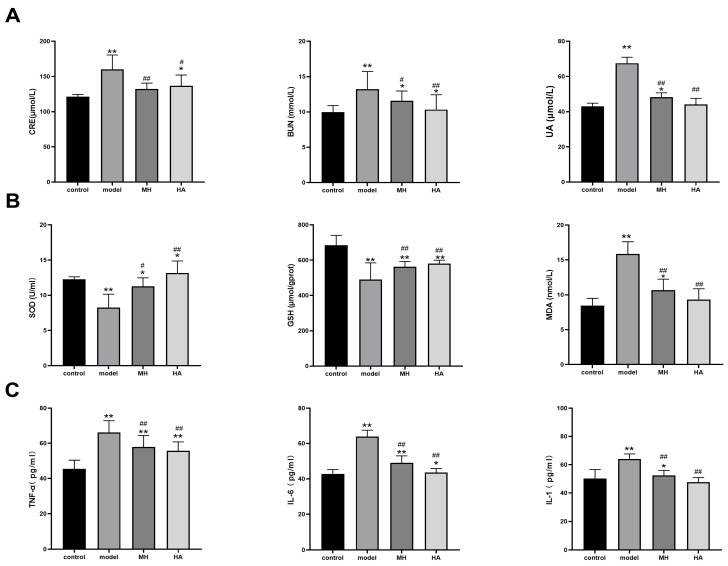
Effects of HA on renal function, antioxidant level, and inflammatory factors. (**A**) The CRE, BUN, and UA test results. (**B**) The SOD, GSH, and MDA test results. (**C**) The TNF-α, IL-6, and IL-1 test results. Values are expressed as mean ± SD (*n* = 6). * *p* < 0.05 and ** *p* < 0.01 versus control; # *p* < 0.05 and ## *p* < 0.01 versus model.

According to the findings presented in Figure 8A, it was observed that the serum levels of creatinine (CRE), blood urea nitrogen (BUN), and uric acid (UA) were significantly elevated (*p* < 0.01) in the model group compared to the normal group, suggesting the presence of renal tissue damage. Conversely, the levels of serum CRE, BUN, and UA were found to be reduced (*p* < 0.05) in both the MH group and HA treatment group compared to the model group, indicating a similar intervention effect of HA and salt MH drugs. These results provide evidence that T2DM can lead to kidney damage and subsequently result in diabetic nephropathy (DN), while AR demonstrates efficacy in preserving renal function.

#### 2.7.3. Effects of Oxidative Damage Index and Inflammatory Markers in Kidney Tissues

The findings of this study indicate that the levels of SOD and GSH were significantly lower in the model group compared to the control group, while the levels of MDA were significantly higher. Conversely, the levels of SOD and GSH were higher in the MH group and HA group compared to the model group, while the level of MDA was lower, particularly in the HA group. These results suggest that HA has the potential to enhance antioxidant stress levels in kidney tissue of DN mice, as illustrated in Figure 8B.

Furthermore, through the utilization of an ELISA kit for the examination of rat serum, it was observed that the concentration of inflammatory factors in the experimental group exhibited a noteworthy elevation in comparison to the control group (*p* < 0.01). Moreover, subsequent to the administration, a reduction in the serum levels of TNF-α, IL-6β, and IL-1 was observed in contrast to the experimental group (*p* < 0.01), thereby suggesting that AR possesses anti-inflammatory properties, as depicted in Figure 8C.

#### 2.7.4. Histopathological Examination of Kidney

**Figure 9:**
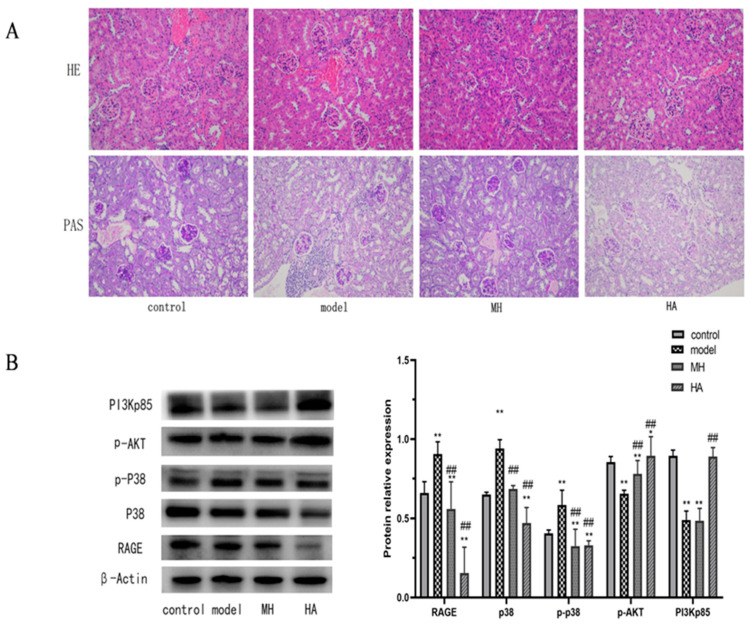
The results of pathological observation and Western blot. (**A**) H&E staining and PAS staining show the typical images of renal pathological structure in each group (magnification: 200×). (**B**) Western blotting is used to detect relative protein expression levels in liver tissue. Values are expressed as mean ± SD (*n* = 3). * *p* < 0.05 and ** *p* < 0.01 versus control; ## *p* < 0.01 versus model.

Based on the findings from the H&E staining analysis (Figure 9A), it can be observed that the control group exhibits intact glomeruli and renal tubules, characterized by well-organized epithelial cells with preserved morphology and uniform cytoplasm. Conversely, in the model group, degeneration and necrosis of glomerular endothelial cells were evident, along with the presence of vacuoles of varying sizes in the renal tubular epithelial cells. Additionally, a minority of renal tubular epithelial cells exhibited necrosis and exfoliation, accompanied by pronounced inflammatory infiltration. The glomerular morphology appeared normal in both the MH group and HA group, with a reduction in inflammatory infiltration. Furthermore, the PAS group observation revealed heavier glycogen staining in the kidney tissue of mice in the model group, indicating the deposition of glycogen in the kidney. The glycogen staining appeared purple–blue and positive but became lighter after administration.

#### 2.7.5. Effect of HA on the Expression of RAGE, p38, p-AKT, and PI3Kp85 Protein in Kidney Tissue of Model Mice

The results depicted in Figure 9B demonstrate significant differences in the expression levels of RAGE, p38, p-P38, p-AKT, and PI3Kp85 between the model group and the control group (*p* < 0.01). Following treatment, there was a significant decrease in the expression levels of RAGE, p38, and protein (*p* < 0.01), while the expression levels of p-AKT and PI3Kp85 protein exhibited a significant increase (*p* < 0.05). Notably, the expression level of HA histone was significantly higher than that of the model group (*p* < 0.01) and more pronounced than that of MH. These findings suggest that HA exerts a notable regulatory effect on these proteins, warranting further investigation.

## 3. Discussion

Diabetes mellitus is a metabolic disorder characterized by elevated blood glucose levels, and it is recognized as the third-most prevalent non-communicable disease that significantly impacts both physical and mental well-being. It ranks below malignant tumors and cardiovascular diseases in terms of the severity of its effects. Of particular concern is the increasing burden of Type 2 diabetes mellitus, which poses a major challenge to global healthcare systems [15]. 

We conducted enzyme activity assays to evaluate the inhibitory effects of AR extracts on key enzymes involved in glucose metabolism. The results demonstrated significant inhibitory activity, suggesting the presence of potent hypoglycemic components in AR. In our study, we initially employed enzyme activity to screen the hypoglycemic active components of AR, followed by the utilization of UPLC-QE-Orbitrap-MS for compound composition identification. After considering the aforementioned findings, we conducted an extensive network pharmacological analysis to predict the targets. Subsequently, our predicted active components and targets were confirmed by molecular docking and related experimental procedures.

A total of 23 compounds were present in HA, the active constituent of AR, and were subjected to analysis using UPLC-QE-Orbitrap-MS. Subsequently, network pharmacology was employed to elucidate the principal components and targets of AR in the management of T2DM. It is noteworthy that flavonoids, namely, methylnissolin, calycoside-7-O-*β*-D-glucoside, and formononetin, have been identified as the primary active components of AR with significant hypoglycemic properties. These flavonoids exhibit remarkable attributes, including anti-apoptotic, anti-inflammatory, and therapeutic effects on renal injury and renal fibrosis [16,17,18]. The key targets in this study included PIK3CA, AKT1, MAPK1, and TNF. AKT1, as the primary downstream target of the PI3K-Akt signaling pathway, plays a crucial role in facilitating the transportation of insulin to various tissues such as the liver, pancreas, skeletal muscle, and adipose tissue. This transportation process is essential for maintaining glucose homeostasis, promoting insulin secretion, and regulating lipid metabolism balance, and PIK3CA specifically plays a critical role in insulin metabolism and blood sugar regulation [19,20]. MAPK1 is associated with numerous inflammatory pathways and serves as a multifunctional cytokine implicated in the proliferation, differentiation, and functional modulation of diverse cell types. It assumes a pivotal role in immune and inflammatory responses, while also exhibiting the ability to impede insulin resistance in individuals with T2DM [21]. The enrichment analysis of the KEGG pathway demonstrated that the differential targets were primarily concentrated in signaling pathways, including the AGE-RAGE signaling pathway in diabetic complications, Type 2 diabetes mellitus, and the PI3K-Akt signaling pathway. 

To corroborate the preceding prognostic outcomes, we implemented a T2DM mouse model by employing STZ in conjunction with high-fat feeding [22,23]. The analysis of renal function indices revealed significant disparities between the model group and the control group, with the latter exhibiting relatively elevated blood sugar levels, thereby substantiating the success of the experimental model. Furthermore, the alterations in biochemical indices and blood sugar levels in the model mice demonstrated that the administration effectively regulated blood sugar levels over time, resulting in a substantial decrease in the levels of renal function indices such as creatinine (CRE), blood urea nitrogen (BUN), and uric acid (UA). The concentrations of superoxide dismutase (SOD) and glutathione (GSH) in renal tissue exhibited an increase, whereas the levels of malondialdehyde (MDA) demonstrated a decrease. Additionally, the levels of tumor necrosis factor-alpha (TNF-α), interleukin-6 beta (IL-6β), and interleukin-1 (IL-1) inflammatory factors were inhibited. Creatinine (CRE), blood urea nitrogen (BUN), and uric acid (UA) serve as crucial indicators for assessing renal function, effectively reflecting the renal and glomerular filtration functions of the body. Deviations from normal values of these indicators indicate impaired renal function [24]. Superoxide dismutase (SOD), glutathione (GSH), and malondialdehyde (MDA) primarily serve as indicators of the body’s antioxidant stress level. SOD and GSH exhibit antioxidant properties and contribute to anti-aging effects. In instances where the kidney generates an excessive amount of superoxide and hydroxyl radicals, the redox equilibrium becomes disrupted, resulting in diminished levels of SOD and GSH and increased levels of MDA, along with diminished tissue antioxidant capacity being observed [25,26]. Inflammatory cytokines TNF-α and IL-6 are swiftly and transiently produced in response to infection and tissue injury [27]. Both IL-1β and IL-6 serve as inflammatory mediators associated with inflammation. Activation of IL-1β has been shown to stimulate the NF-κ B signaling pathway and enhance the secretion of IL-6 [28]. These findings provide evidence that AR not only reduces blood glucose levels in model mice, but also ameliorates renal tissue damage induced by T2DM.

Furthermore, the outcomes of histological examination using H&E and PAS staining revealed that the integrity of glomerular and renal tubular structures was compromised in the diabetic model group, accompanied by an elevation in polysaccharide deposition. This can be attributed to the induction of hyperglycemia, which subsequently triggers excessive glucose uptake, resulting in glomerular impairment and aberrant renal function [29]. The findings indicate that the active constituents of AR exhibit specific effects in ameliorating renal damage in individuals with T2DM.

**Figure 10:**
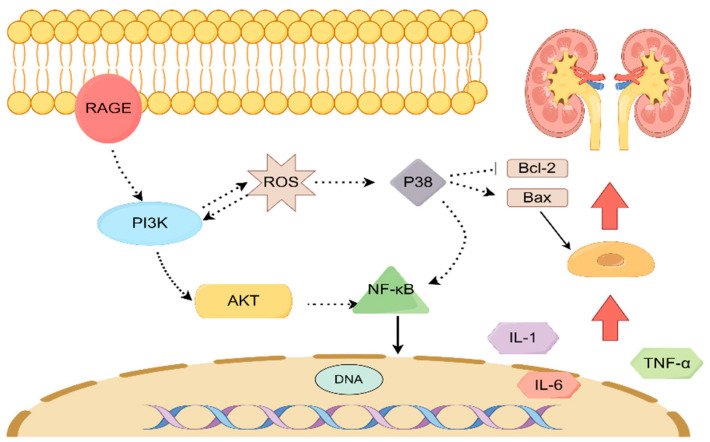
The mechanism of action of AR in the treatment of T2DM renal injury.

To enhance our comprehension of the therapeutic mechanism of AR in the management of T2DM, we conducted an analysis of the protein blot to assess the expression levels of RAGE, p38, p-P38, p-AKT, and PI3Kp85 proteins. This investigation was conducted in conjunction with the KEGG analysis results. The AGE-RAGE signaling pathway in diabetic complications is a crucial signal transduction pathway wherein protein glycosylation products resulting from hyperglycemia bind to their corresponding RAGE receptors, thereby initiating a cascade of reactions. This signaling pathway assumes a significant role in the initiation and progression of diabetic complications [30]. The protein RAGE, which plays a crucial role in the development of Type 2 Diabetes Mellitus (T2DM) and various other disorders, demonstrates the ability to regulate multiple pathways, such as the MAPK family, PI3K-AKT, and several others [31]. RAGE is predominantly present in glomerular epithelial cells, renal tubular epithelial cells, and glomerular membrane cells within the kidney. Its presence in these cells enables the regulation and mitigation of inflammatory infiltration and fibrosis in diabetic mice [32]. P38 is primarily accountable for the regulation of inflammation and apoptosis within the MAPK signaling pathway [33,34]. The existing literature suggests that the reduction of P38 and the activation of Akt and NFκB significantly contribute to the protective impact of pharmaceutical interventions on renal ischemia-reperfusion injury in rats [35]. PI3K is an enzyme consisting of a heterodimeric structure, comprising the catalytic subunit p110 and either the non-catalytic subunit p85 or p110. Extensive research has revealed a direct correlation between the PI3K/Akt signaling pathway and the development of diabetic nephropathy, highlighting it as a crucial target in this context [36]. In the transduction pathway of insulin effect, any disruption in the PI3K/Akt signaling pathway may result in insulin resistance [37]. Furthermore, it has been reported that the activation of Akt has the capability to transmit biological signals to receptor substrates downstream, thereby initiating a range of biological effects such as glucose absorption, glycolysis, glycogen synthesis, and protein synthesis [38]. Hence, it is postulated that the potential mechanism underlying the therapeutic effects of AR in the treatment of T2DM involves the activation of the RAGE protein, subsequently modulating the pivotal PI3K/Akt pathway associated with renal injury (see Figure 10).

## 4. Materials and Methods

### 4.1. Materials and Reagents

Goat anti-rabbit antibody was acquired from Utibody (No. UT200). Streptozocin (STZ) was obtained from Beijing Solarbio Science and Technology Co., Ltd. (Beijing, China). A high-fat diet (HFD; 47% carbohydrate, 28% fat, 25% protein) was purchased from Changchun Yisi Experimental Animal Technology Co., Ltd. (Changchun, China). The α-glucosidase enzyme was procured from Sigma Company. Acarbose was purchased from Shanghai Yuanye Biotechnology Co., Ltd. (Shanghai, China). RAGE (No. WL01514) and p-P38 (No. WLP1576) were obtained from Wanlaibao (Foshan, China). P-AKT (No. YT0185), P38 (No.YT3511), PI3K-P85 (No. YM3503), and anti-β-actin antibodies (No. YM3028) were sourced from Immunoway (Plano, TX, USA).

### 4.2. Plant Materials and Extraction

AR was obtained from Jilin Pharmacy Co., Ltd., located in Changchun, China. The AR was pulverized and was then decocted with eight times the amount of water for 2 h each time. The resulting decoction was collected and divided into two parts. The decocted components were filtered by an ultrafiltration system with a regenerated cellulose composite membrane (Ultracellulose PLBC, Millipore, MA, USA) with a molecular weight of 3 kDa, and two membrane separation components were obtained: fraction A (HA) with a molecular weight less than 3 kDa and fraction B (HB) with a molecular weight greater than 3 kDa. The precipitate fraction C (HC) and supernatant fraction D (HD) were obtained by water extraction and alcohol precipitation. These four fractions were then evaporated in a water bath maintained at 80 °C and stored at 4 °C for future use.

### 4.3. Measurement of α-Glucosidase Inhibitory Activity

The reference method underwent a slight modification [39]. In a 96-well plate, 80 μL of 0.1 mol/L phosphate buffer (pH 6.8) was dispensed, followed by the addition of 20 μL of the sample solution and 100 μL of α-glucosidase (0.5 U/mL) solution. The plate was then incubated in a shaker at 37 °C for 20 min. Subsequently, 50 μL of PNPG (5 mmol/L) solution was added. The absorbance A was measured at 405 nm at 0 min and 5 min using an enzyme-labeled instrument and the IC50 value was calculated. Each sample was subjected to at least three parallel measurements and the average value was determined. The formula is:

Suppression ratio% = {[(A_5 min_ − A_0 min_) control – (A_5 min_ − A_0 min_) sample]/(A_5 min_ − A_0 min_) control} × 100%

### 4.4. UPLC-QE-Orbitrap-MS Condition

The Poroshell 120 EC-C18 column (250 mm × 4.6 mm, 4.0 μm) was used with a flow rate of 0.5 mL/min and a column temperature of 35 °C. The mobile phase consisted of a 0.1% formic acid aqueous solution (mobile phase A) and acetonitrile (mobile phase B). Gradient elution was performed with a range of 0–20 min at 2–49% B, 20–30 min at 49–90% B, and 30–55 min at 90–100% B. The analysis was conducted in electrospray negative ion scanning mode (ESI-) with a dry gas (N2) flow rate of 9 L/min, a temperature of 300 °C, an atomizing gas pressure of 2.41 × 10^5^ Pa, a capillary voltage of 3.5 kV, a crushing voltage of 175 V, a cone voltage of 65 V, and a mass scanning range of *m*/*z* 66.5 to 1000.

### 4.5. AR Treatment of T2DM Target Acquisition

The HA components were subjected to analysis using the PubChem (https://PubChem.ncbi.nlm.nih.gov/ (accessed on 14 May 2023)) and SwissTargetPrediction (http://swisstargetprediction.ch/ (accessed on 14 May 2023)) databases in order to predict the target of these components. The search for T2DM-related disease targets was conducted through the DrugBank database, OMIM database, and GeneCard database. The GeneCard database employed a selection criterion of targets with Relevance scores above the average value, and these targets were combined to eliminate any duplicated target genes, resulting in the identification of disease-related targets. Subsequently, these disease-related targets were matched with the active ingredient target to determine the potential for treating T2DM.

### 4.6. KEGG Pathway Analysis

The target should be entered into the Metascape database (https://Metascape.org/ (accessed on 2 June 2023)) to conduct functional enrichment analysis and visualization of Kyoto Gene and Genome Encyclopedia (KEGG). The species “Homo sapiens” should be chosen for this purpose.

### 4.7. Network Construction of “Component-Core Target-Signaling Pathway” 

The active ingredient A, along with the core target and signaling pathway data, were imported into Cytoscape 3.7.2 software for network construction. The topological parameters were subsequently analyzed using the Network Analyzer plug-in.

### 4.8. Molecular Docking

The active compounds were searched by the Pubmed database and the SDF (*.sdf) format files of the compounds were downloaded and converted into the pdbqt (*. Pdbqt) format. The PDB database was used to retrieve and download the core target protein obtained by the Cytoscape software. Molecular docking was carried out by using Autodock Vina 1.5.6 software.

### 4.9. Molecular Dynamics Simulation

The initial conformation of molecular dynamics simulation is obtained by the results of molecular docking. Gromacs, a dynamic simulation software, is used and Charmm36 force field and TIP3P water model are used. Establish a water tank and add a sodium ion balance system. During the simulation operation, the energy minimization of the whole system is performed to achieve better molecular configuration. Subsequently, the system is balanced under canonical ensemble (NVT) and isothermal–isobaric ensemble (NPT) at ambient temperature and pressure and is used for 100 ns molecular dynamics simulation. RMSD, RMSF, and protein skeleton radius of gyration are used to further evaluate the simulated trajectory.

### 4.10. Animals Experiment In Vivo

#### 4.10.1. Animals and Experimental Design

Fifty male SPF mice weighing 20 ± 3 g were obtained from Changchun Yisi Experimental Animal Technology Co., Ltd. This study received approval from the Animal Protection and Use Committee of Changchun University of Chinese Medicine (No. 2023447). The mice were housed in a controlled environment at approximately 22 ± 2 °C, with a 12 h light/dark cycle, and were provided with unrestricted access to standard food and tap water. 

Mice with fasting blood glucose levels below 6.00 mmol L^−1^ were randomly assigned to four groups: control group, model group, metformin hydrochloride (MH) group (150 mg/kg body weight), and HA group (150 mg/kg body weight). Each group consisted of 10 mice. The control group was fed a regular diet, while the other groups were fed a high-fat diet. Starting from the fifth week, the mice were intraperitoneally injected with 60 mg/kg of streptozotocin (STZ), which was dissolved in 0.05 mol/L sodium citrate with a pH of 4.5 prior to injection. Simultaneously, the mice in the control group received intravenous injections of an equivalent volume of 0.9% NaCl aqueous solution for a duration of 5 days, thereby establishing the model. The criterion for model success was defined as a blood sugar value exceeding 9.0 mmol/L. Subsequently, the drug was administered continuously for a period of 4 weeks, and daily monitoring of blood glucose levels in the tail vein was conducted using a blood glucose meter.

The animals were euthanized 12 h after the final administration, and subsequent blood and tissue samples were obtained. A portion of the samples were preserved at a temperature of −80 °C in a refrigerator, while the remaining specimens were stored in a 4% formalin buffer for subsequent histological analysis.

#### 4.10.2. Analysis of Serum and Tissue

The blood urea nitrogen (BUN), creatinine (CRE), and uric acid (UA) kits were acquired from Nanjing JianCheng Institute of Biological Engineering in Nanjing, China. Superoxide dismutase (SOD), glutathione (GSH), and malondialdehyde (MDA) enzyme-linked immuno-sorbent assay (ELISA) kits were obtained from Changchun Bestgene Biotechnology Co., Ltd. in Changchun, China. Enzyme-linked immuno-sorbent assay (ELISA) kits for tumor necrosis factor-α (TNF-α), interleukin-6 (IL-6), and interleukin-1 (IL-1) were purchased from American R&D. All operational procedures were meticulously followed in accordance with the manufacturers’ instructions for the utilized kits.

#### 4.10.3. Histopathological Examinations

The tissue specimen was initially preserved in a 10% neutral paraformaldehyde solution, subsequently embedded in paraffin, manually sectioned using a microtome to achieve 4 μm thick slices, subjected to dewaxing and hydration processes, stained using the H&E and PAS techniques, and finally examined under an optical microscope (Nikon, Tokyo, Japan).

#### 4.10.4. Western Blotting

The quantification of total protein extracted from renal tissue was performed using the BCA detection kit (Solaibao, Beijing, China). A 10% separation gel and 5% concentrated gel were prepared, and the samples were loaded and subsequently electro-transferred onto nitrocellulose membranes. The wet transfer of the membrane onto an NC/PVDF membrane (0.22 µm, Millipore, Burlington, MA, USA) was conducted for a duration of 1 h. Afterwards, the membrane was obstructed using 5% skimmed milk powder for a duration of 1 h, and subsequently subjected to incubation with the primary antibody overnight at a temperature of 4 °C. Following this, the membrane was rinsed and exposed to the second antibody at room temperature. The gray values were then documented subsequent to electrochemiluminescence development. Result analysis was conducted using Gel-Pro Analyzer 4 (Media Cybernetics, Rockville, MD, USA).

### 4.11. Statistical Analysis

All samples were subjected to a minimum of three evaluations, with the resulting values representing the average standard deviation (SD). The disparities between groups were assessed through the utilization of one-way ANOVA and back testing (SPSS 22.0). Graphics were generated using GraphPad Prism 8.0. *p* < 0.05 indicates a significant difference and *p* < 0.01 indicates an extremely significant difference.

## 5. Conclusions

In summary, the utilization of enzyme activity, network pharmacology, molecular docking, molecular dynamics, and in vivo experiments revealed the significant contribution of flavonoids present in AR to the therapeutic management of T2DM. AR exhibits antioxidant and anti-inflammatory properties that mitigate renal damage induced by T2DM, while also modulating the RAGE/PI3K/Akt signaling pathway to lower blood glucose levels and enhance renal function. These findings establish a solid foundation for future investigations into the potential clinical utilization of AR for T2DM treatment.

## Figures and Tables

**Table 1:** MS data for characterization of compounds in HA by UPLC-QE-Orbitrap-MS.

No.	Time	Formula	Measured Weight	MS^2^ Characteristic Ions (*m*/*z*)	Chemical Compound
**1**	3.8	C_22_H_22_O_12_	477.1027	253.0930, 150.9785	Isorhamnetin-3-Glucoside
**2**	3.84	C_5_H_11_NO_2_	188.0347	58.0657	Betaine
**3**	4.10	C_16_H_18_O_9_	353.0709	191.0189, 179.0547, 161.0440	Chlorogenic acid
**4**	4.19	C_15_H_10_O_7_	303.0414	119.0333, 117.0174, 151.0972	Quercetin
**5**	9.05	C_10_H_13_N_5_O_4_	268.1028	268.1036, 136.0617	Adenosine
**6**	9.86	C_15_H_10_O_5_	271.1024	254.1129	Apigenin
**7**	10.81	C_9_H_11_NO_2_	166.0856	166.0860, 120.0808	Phenprobamate
**8**	12.97	C_23_H_24_O_10_	461.1396	299.0885	6,4′-dimethoxyisoflavone-7-*O*-glucoside
**9**	13.30	C_16_H_12_O_6_	299.0764	285.0621, 239.0517	Kaempferide
**10**	15.87	C_9_H_11_NO_3_	182.0780	164.1181, 136.0012, 119.9608	Tyrosine
**11**	16.64	C_22_H_22_O_10_	447.1263	285.0753, 270.0517, 253.0484, 225.0542	Calycosin-7-*O*-*β*-D-glucoside
**12**	17.90	C_16_H_12_O_5_	285.0330	270.0518, 253.0490, 225.0544, 137.0232	Calycosin
**13**	18.59	C_22_H_22_O_9_	431.1172	269.0414	Ononin
**14**	20.90	C_17_H_18_O_5_	303.1209	193.0855, 181.0853, 167.0701, 123.0441	7,2′-Dihydroxy-3′,4′-dimethoxyisoflavan
**15**	21.65	C_25_H_24_O_12_	514.3209	187.0961, 225.1126	Malonyl ononin
**16**	22.71	C_17_H_14_O_6_	315.0844	300.0619	Odoratin
**17**	23.14	C_24_H_24_O_10_	473.1461	270.0837, 269.0804	Formononetin-7-*O*-*β*-Dglucoside-6″-Oacetate (4), (8)
**18**	23.70	C_14_H_16_N_2_O_5_	293.1343	165.0365, 135.0803	Methylnissolin
**19**	24.08	C_6_H_14_O_2_N_4_	175.0695	116.3644	L(+)-Arginine
**20**	25.33	C_11_H_12_N_2_O_2_	205.6131	163.8078, 149.0228	D(+)-Tryptophan
**21**	26.77	C_16_H_12_O_4_	269.0730	254.0569, 237.0544, 226.0612, 118.0414	Formononetin
**22**	27.42	C_16_H_12_O_6_	301.1030	286.0821, 241.0858	Pratensein
**23**	34.22	C_18_H_32_O_3_	295.1392	195.1378, 171.1011	13-Hydroxy-9,11-octadecenoic acid

**Table 2:** Blood sugar values of mice in each group after administration (mmol·L^−1^).

Group	0 d	7 d	14 d	21 d	28 d
Control	5.23 ± 0.21	5.30 ± 0.35	5.12 ± 0.26	5.27 ± 0.21	5.17 ± 0.23
Model	10.02 ± 1.01	10.93 ± 0.32	11.9 ± 0.30	12.93 ± 0.25	12.09 ± 1.10
MH	9.60 ± 0.81	9.36 ± 0.46	8.62 ± 0.93 *	7.53 ± 1.22 **	6.6 ± 0.85 **
HA	9.54 ± 0.76	8.3 ± 1.06 *	7.85 ± 0.93 *	6.76 ± 1.13 **	5.7 ± 1.47 **

Values are expressed as mean ± SD (*n* = 6); * *p* < 0.05 and ** *p* < 0.01 versus model.

## Data Availability

Data are contained within the article.
